# Supplementary material for: Corporate governance and reporting quality of accounts in China-listed firms. A moderating role of ownership pattern
Source: PLoS One. 2023 Nov 30;18(11):e0295253. doi: 10.1371/journal.pone.0295253 (PMC10688956; doi:10.1371/journal.pone.0295253)
Supplement: Attachment — Submitted filename: Response to reviewers.docx [file pone.0295253.s003.docx]

Review response by author

**Reviewer #1:**

Dear authors, Thank you so much for your effort. The manuscript was very good prepared and designed. However, I have one main concern which is about the hypotheses. You have presented 14 hypotheses but i did not see the tests of the same 14 hypotheses if they are accepted or rejected. The authors should add one main table including all the hypotheses and why each hypothesis statistically accepted or rejected. Al the best.

**Response to Reviewer 1.** Hypothesis are summarized and the table added for the accepted or rejected and also the reason is written. The changes are shown in highlights in the main manuscript.

**Reviewer #2:**

I have reviewed the article and I am of the view that the article needs lot of improvement. The following points need consideration:

1. The research gap is not clear.
2. The reason that why China is considered research ground is missing.
3. Grounding of hypotheses is not appropriate.
4. The authors have discussed the agency theory and steward theory. These theories need to be embedded in hypotheses development and results.
5. Variable measurement is not appropriate. The board independence should be measured using proportion of independent directors.
6. None of the correlation coefficient is significant.
7. The results are not discussed in the light of literature.

**Response to Reviewer 2.** The research gap makes clearer. The reason of the China selection with background are stated. Hypotheses are properly ground. The theories are discussed in hypothesis and results. Variables measurement make appropriate. Correlation coefficient discussed. The results are also discussed with previous literature. The yellow highlighted shows the corrections on the recommendations of the reviewer.

**Reviewer #3:**

The current work is new topic and cater ongoing issues among the academics and policy makers. However, I have following suggestions, which can further improve the quality of the manuscript. 1. There is large number of Hypothesis….. 14 hypothesis mentioned ….I would suggest to club some the hypothesis as possible……

2. The authors used non-financial firms for the study sample…. I would suggest add some justification.. why you exclude the financial sector of the economy.

3. Page 17, “China on the basis of random sampling that has the greater market capitalization from the 1000 top market capitalized firms” mentioned by author with reference…… I am unable to understand about this…. I would suggest to add more explanation.

4. The explanation/ Definition of the depended variable i.e. Auditor lag is missing….. Throughout the paper… Also missing in the Table 3.1.

5. The correlation matrix fail to discuss the significance of the correlation coefficient among the variables. My suggestion to mentioned the significance level with correlation coefficients. 6. I would suggest to club the Table 4.3 and Table 4.4 into one table…. Which, may improve the quality and reduced the length of the paper…. Further, It looks that the current manuscript is extracted from Research thesis/project. Usually, the Table 4.1, 4.3… 2.1 not used research articles. Suggest to revisit the table numbering…….

**Response to Reviewer 3:** The hypothesis is clubbed according to your suggestions. The reasons of the non-financial sector selection are given. The selection of the China firms as sample reason explained also. Auditor lag explanation is given. correlation matrix explained with coefficients. Table are numbered according to the reviewer suggestions. The highlights show the changes.

***Review response on Editor comments***

1. File name are changes as the editor suggestions.
2. Funding information’s provided in the online submission.
3. Correct the financial disclosure information with the role of funder.
4. Removed the funding information from the manuscript successfully.
5. Competing interest statement added in cover letter.
6. Data availability statement added.
7. ORCID provided of the author.
8. Figure referred in the text.
9. All the tables and figures text in the manuscript that helpful for production.
