# Supplementary material for: Corporate governance and reporting quality of accounts in China-listed firms. A moderating role of ownership pattern
Source: PLoS One. 2023 Nov 30;18(11):e0295253. doi: 10.1371/journal.pone.0295253 (PMC10688956; doi:10.1371/journal.pone.0295253)
Supplement: Attachment — Submitted filename: Report.docx [file pone.0295253.s004.docx]

| 1. | Introduction | 1. The scholar has presented quite in-depth details about the Corporate governance, FRQ and a very good approach is used to address the problem.  2. Formatting, check spelling, punctuations and grammar issue.  3. Research objectives and questions are missing. |
| --- | --- | --- |
| 2. | LITERATURE Review | 1. Part 2 starts with a systematic approach and provides thorough literature review, but theoretical support must be more vital to justify the topic. 2. The candidate has set the several hypothesizes from H1, H2, H3, H4 and H5 .   For example H1: Board characteristics of corporate governance have significant positive relationship with reporting quality of financials.  For hypothesis testing , if the outcome is yet to be known, it is sufficient to mentions positive impact  (drop the significant word).   3. The candidate should demonstrate how the research relates to the literature. The research findings and discussion should be linked with the literature and theoretical grounds for the conducted research in a better way. |
| 3. | Methodology | 1. Appropriate research methods have been used. The research methodology is acceptable to justify the result. 2. Mention the proper approach of moderating technique. |
| 4 | Analytical tool | i. Why descriptive statistic important for your data? ii. In this analysis part , researcher need t explain that how and why your selected sampling needs descriptive analysis and correlation analysis. iii. Mention recent studies for ownership structure as moderating variable. |
| 5. | Conclusion | After all of the analysis is completed , each objective should be highlighted again in the conclusion chapter by providing discourse demonstrating where the analysis is leading the work and what the outcome is, and then linking that to literature reviews. |
| 6 | References | Please check the references again and references are not as per APA format that requires attention. |
