# Supplementary material for: Corporate governance and reporting quality of accounts in China-listed firms. A moderating role of ownership pattern
Source: PLoS One. 2023 Nov 30;18(11):e0295253. doi: 10.1371/journal.pone.0295253 (PMC10688956; doi:10.1371/journal.pone.0295253)
Supplement: Attachment — Submitted filename: reviewe.docx [file pone.0295253.s006.docx]

Comments to the Author

This work investigates “Corporate governance and reporting quality of accounts in China listed firms. A moderating role of Ownership pattern.” I am reasonably familiar with the literature on the topic and have ongoing research interest in this subject matter. I believe the topic of the research is quite interesting as the current draft attempts to unfold some hidden layers which are yet not been tackled by the previous scholars. Below, I list and comment on the findings presented:
1. The abstract should be short,” and these information: "What does the author(s) want to know? (Purpose): Why do they want to know? (gap) How do they want to know? (Methodology): What are the outcomes? (Results): What are the developments of the study? (Contribution): What are the limitations of the study? (future research direction)” must be included in the abstract. The rest of the irrelevant information should be deleted.
2. I believe the introduction is quite comprehensive. Anyway, the following information should be added at the second last paragraph in the introduction section: (a) The introduction should underline the aim of the paper; (b) stress why this aim is important (with the support of the literature); (c) summarize the methodology; and (d) describe the development of the study.
3. In my opinion, this section needs to be developed a bit. The authors have not critically reviewed the existing works.
4. The methodology should be explained properly so that the readers can understand it easily. Operational definition of variables must be presented in appropriately. Data sources must be defined. Sampling technique used must be justified.
5. Discussion should include comparing and contrasting with previous studies, discussion of the results, theoretical contribution, practical implication, and future research direction. Results should include diagnostic statistics presented in appropriate manner.
6. The conclusion should be separated from the discussion. Policy implications should be provided in light of findings of study.


Good Luck
