# Supplementary material for: Corporate governance and reporting quality of accounts in China-listed firms. A moderating role of ownership pattern
Source: PLoS One. 2023 Nov 30;18(11):e0295253. doi: 10.1371/journal.pone.0295253 (PMC10688956; doi:10.1371/journal.pone.0295253)
Supplement: Attachment — Submitted filename: Responce to Editor and reviewer Comments.docx [file pone.0295253.s005.docx]

**Editor Comments:**
I have reviewed the article entitled “Corporate governance and reporting quality of accounts in China listed firms. A moderating role of Ownership pattern”. This is an interesting study and the authors have collected a unique dataset for a unique and progressive methodology. The paper is generally well written and structured. However, in my opinion the paper has some shortcomings in regard to some data and text, and I feel this unique dataset has not been utilized to its full extent. Below I have provided numerous remarks on the text:
1. In several instances, I suggest citing more relevant and recent studies in the introduction and methodology sections.

***Response to point 1:***

*The most recent and relevant studies are cited successfully in the introduction and methodology section according to the suggestions of the editor.*

2. The introduction should be expanded to include importance, uniqueness and contribution to the literature.

***Response to point 2:***

*Introduction makes more attractive that includes the importance uniqueness and contribution to the literature. Contribution of the study are written successfully.*

3. You need to state clearly the contributions of the paper. For example, "Consequently, the current paper seeks to make the following contributions to the existing literature. First,…, Second,…., Third, …, Fourth,… and so on". The description of the contribution needs to be more forensic, needs to be more focused.

***Response to point 3:***

*The contribution makes according to editor suggestions. Please find green highlighted page 6 in the introduction as*

*“This study makes significant contributions to the existing literature and theories on corporate governance and financial reporting quality. Firstly, it advances the development and refinement of established theories like agency theory, stewardship theory, and signaling theory. By exploring the connections between corporate governance mechanisms and financial reporting quality, the study identifies and elucidate the underlying processes and mechanisms that drive these relationships in various industrial and cross-cultural settings worldwide. Secondly, ………………………………………Fourth, it contributes about the moderating impact of the family ownership and ownership concentration in the emerging markets, ………………………. Furthermore, this study help identify similarities and differences in corporate governance practices and their impact on financial reporting quality by examining the relationships between corporate governance and financial reporting quality in various countries and contexts”.*

4. The authors should discuss the relevant theories in detail and relate their findings to a specific theory of corporate governance and Ownership pattern in the developing country context

***Response to point 4:***

*The relevant theories in context of the developing economy are discussed in the literature review and findings are also discussed with support the findings. The green highlighted page 6-7 shows the track changes.*

5. There are multiple misspellings throughout the manuscript that need attention.

***Response to point 5:***

*The spellings and other grammatically mistakes removed from the manuscript.*

**Reviewer #4:**

Introduction
1. The scholar has presented quite in-depth details about the corporate governance, FRQ and a very good approach is used to address the problem.
2. Formatting, check spelling, punctuations and grammar issue.
3. Research objectives and questions are missing.

***Response to section Introduction:***

*The Grammarly, misspelling and punctuation error removed. The research objective and questions included in the introduction. Please find page 3-4 yellow highlighted as*

*“*The main objective of this research is to assess the impact of organizational characteristics and corporate governance practices on the accuracy of financial reporting for companies listed in China. The study will specifically examine how ownership concentration and inter-organizational corporate governance (including the board of directors, audit committee, and CEO duality) are related to the reliability of financial reporting. Along with the primary goal, the study has several other specific objectives. Firstly, it aims to evaluate the current state of financial reporting quality in China. Secondly, …………………. Third, …………………. The quality of financial reports will be measured by analyzing the audit reports from the annual financial reports of listed firms.”

LITERATURE Review
1. Part 2 starts with a systematic approach and provides thorough literature review, but theoretical support must be more vital to justify the topic.
2. The candidate has set the several hypothesizes from H1, H2, H3, H4 and H5 .
For example H1: Board characteristics of corporate governance have significant positive relationship with reporting quality of financials.
For hypothesis testing , if the outcome is yet to be known, it is sufficient to mentions positive impact
(drop the significant word).
3. The candidate should demonstrate how the research relates to literature. The research findings and discussion should be linked with the literature and theoretical grounds for the conducted research in a better way.

***Response to section Literature Review:***

*The theoretical support was added in the literature review section. Moreover, the significant words removed from the hypothesis. Please find page 8-11 yellow highlighted. The findings are related to literature and theory in the discussion in detail (please find page 23-27 and yellow highlighted).*

Methodology
1. Appropriate research methods have been used. The research methodology is acceptable to justify the result.
2. Mention the proper approach of moderating technique.
Analytical tool
i. Why descriptive statistic important for your data?
ii. In this analysis part , researcher need t explain that how and why your selected sampling needs descriptive analysis and correlation analysis.
iii. Mention recent studies for ownership structure as moderating variable.

***Response to section Methodology:***

*The moderating techniques used in the analysis are defined in the methodological section also. The data description and correlation analysis reasons are written (please find page 15 and yellow highlighted). Moreover, the recent studies on moderating variables are also included (please find page 10 and yellow highlighted).*

Conclusion
After all of the analysis is completed , each objective should be highlighted again in the conclusion chapter by providing discourse demonstrating where the analysis is leading the work and what the outcome is, and then linking that to literature reviews.
6 References Please check the references again and references are not as per APA format that requires attention.

***Response to section Conclusion:***

*The conclusion is developed according to the reviewer suggestions by supporting previous theory and literature evidence in conclusion section that justified the findings and make significant contribution of the study. Please find page 23-26 yellow highlighted. All the references are corrected and style APA numbered.*

Thank you.
