# Supplementary material for: Corporate governance and reporting quality of accounts in China-listed firms. A moderating role of ownership pattern
Source: PLoS One. 2023 Nov 30;18(11):e0295253. doi: 10.1371/journal.pone.0295253 (PMC10688956; doi:10.1371/journal.pone.0295253)
Supplement: Attachment — Submitted filename: Responce to reviewer comments.docx [file pone.0295253.s007.docx]

Reviewer #4: 

This work investigates “Corporate governance and reporting quality of accounts in China listed firms. A moderating role of Ownership pattern.” I am reasonably familiar with the literature on the topic and have ongoing research interest in this subject matter. I believe the topic of the research is quite interesting as the current draft attempts to unfold some hidden layers which are yet not been tackled by the previous scholars. Below, I list and comment on the findings presented:
1. The abstract should be short,” and these information: "What does the author(s) want to know? (Purpose): Why do they want to know? (gap) How do they want to know? (Methodology): What are the outcomes? (Results): What are the developments of the study? (Contribution): What are the limitations of the study? (future research direction)” must be included in the abstract. The rest of the irrelevant information should be deleted.

**Response:**

The abstract makes the short and attractive according to the suggestion of the reviewer and removed the unnecessary information as,

“Financial reporting quality is critical for businesses, stakeholders, and government to ensure transparency and accountability. The purpose of this paper is to investigate the relationship between corporate governance, financial reporting quality, and ownership structure as a moderating factor for Chinese stock exchange-listed firms. Quantitative data of 550 listed firms from 2012 to 2022 are collected from the annual reports. For investigating the relationship between variables, panel data analysis with random and fixed effect models is used. Our results show that corporate governance's different attributes such as Auditor brand name, Existence of an audit committee, independent board, family ownership, and profitability have a significant negative impact on the audit report lag that decreases the lags and increases the financial reporting quality in China listed firms. Auditor opinion, Board diligence Board size, and CEO duality have a significant positive impact on the audit report lag that increases the lags and decreases the financial reporting quality of China-listed firms. Furthermore, our findings show that ownership concentration has no moderating effect between corporate governance, different attributes, and financial reporting quality. Family ownership, on the other hand, has a strong moderating effect between corporate governance characteristics and financial reporting quality. However, due to limitations, this study provides the opportunity for future research on corporate governance mechanisms in different cultures and environments. Moreover, this study has some significant implications for investors, policymakers, and government.”

2. I believe the introduction is quite comprehensive. Anyway, the following information should be added at the second last paragraph in the introduction section: (a) The introduction should underline the aim of the paper; (b) stress why this aim is important (with the support of the literature); (c) summarize the methodology; and (d) describe the development of the study.

**Response:**

The aim of the study included in the introduction section. The significance of the aims of the study with support to previous literature are written according to the suggestion (please refer yellow highlighted in the Introduction section). The methodology and development of the study is also written in Introduction according to reviewer suggestions (please refer yellow highlighted in Introduction section).

3. In my opinion, this section needs to be developed a bit. The authors have not critically reviewed the existing works.

**Response:**

The most relevant critical literature review added. (please refer yellow highlighted in Literature review section)

4. The methodology should be explained properly so that the readers can understand it easily. Operational definition of variables must be presented appropriately. Data sources must be defined. Sampling technique used must be justified.

**Response:**

The operational definitions section included references.

“The audit report lag is the number of the days delay the audit report by the auditor to complete their audit report due to the numbers of the errors and non-compliance with the code of the corporate governance mechanism [47]. The total member in the board composition is an essential part of the corporate governance mechanism. The board size represents the number of the directors in making board decisions and taking necessary actions [48]. The independent board represents when there is independent directors involve in the board composition [48]. The CEO duality means that when CEO has the double role as CEO and the Chairman of the board of directors [50]. The numbers of the meeting of the board of directors held during the financial year is the board diligence [53]. ………………………………………………………………………………………………………… The family ownership means the shareholding by one member or family has 50% or more in the respect organization [61]. (page 13 Methodology section)

Data sources is properly defined. Sampling techniques is justifying as,

“In this study, we collected secondary data from the years 2012-2022 from the annual reports of the firms that are listed in China stock markets. The annual reports of selected firms are downloaded from the Shanghai Stock Exchange (SSE) data portal”. (page 11 Methodology section)

“A total of 550 non-financial firms are selected as a sample from the total non-financial sector listed in China. There are 4697 listed firms belonging to the non-financial sector of China that represent our populations for the study. The Sample of 550 firms is collected through random sampling from non-financial sectors of the China-listed firms [21, 45, 46]. (page 11 of methodology)

5. Discussion should include comparing and contrasting with previous studies, discussion of the results, theoretical contribution, practical implication, and future research direction. Results should include diagnostic statistics presented in appropriate manner.

**Response:**

The discussion is written according to the suggestion. The yellow highlighted shows the track changes in the discussion section. The findings of the study justified through previous literature in yellow highlighted.

6. The conclusion should be separated from the discussion. Policy implications should be provided in light of the findings of the study.

**Response:**

The conclusion is separated from the discussion. Policy implication, contribution, limitations and future research suggestions are properly written. The yellow highlighted shows the track changes in the Conclusion section.

Thank you
